# Reorganization of Basolateral Amygdala-Subiculum Circuitry in Mouse Epilepsy Model

**DOI:** 10.3389/fnana.2015.00167

**Published:** 2016-01-14

**Authors:** Dong Liang Ma, Jian Qiang Qu, Eyleen L. K. Goh, Feng Ru Tang

**Affiliations:** ^1^Program in Neuroscience and Behavioral Disorder, Duke-NUS Graduate Medical SchoolSingapore, Singapore; ^2^Department of Neurosurgery, Second Affiliated Hospital of Xi’an Jiaotong UniversityXi’an, China; ^3^Department of Physiology, Yong Loo Lin School of Medicine, National University of SingaporeSingapore, Singapore; ^4^KK Research Center, KK Women’s and Children’s HospitalSingapore, Singapore; ^5^Temasek Laboratories, National University of SingaporeSingapore, Singapore; ^6^Singapore Nuclear Research and Safety Initiative, National University of SingaporeSingapore, Singapore

**Keywords:** reorganization, basolateral amygdala, subiculum, epilepsy, mouse, pilocarpine, contextual fear memory test, c-Fos

## Abstract

In this study, we investigated the reorganized basolateral amygdala (BLA)-subiculum pathway in a status epilepticus (SE) mouse model with epileptic episodes induced by pilocarpine. We have previously observed a dramatic loss of neurons in the CA1–3 fields of the hippocampus in epileptic mice. Herein, we observed a 43–57% reduction in the number of neurons in the BLA of epileptic mice. However, injection of an anterograde tracer, *Phaseolus vulgaris* leucoagglutinin (PHA-L) into the BLA indicated 25.63% increase in the number of PHA-L-immunopositive terminal-like structures in the ventral subiculum (v-Sub) of epileptic mice as compared to control mice. These data suggest that the projections from the basal nucleus at BLA to the vSub in epileptic mice are resistant to epilepsy-induced damage. Consequently, these epileptic mice exhibit partially impairment but not total loss of context-dependent fear memory. Epileptic mice also show increased c-Fos expression in the BLA and vSub when subjected to contextual memory test, suggesting the participation of these two brain areas in foot shock-dependent fear conditioning. These results indicate the presence of functional neural connections between the BLA-vSub regions that participate in learning and memory in epileptic mice.

## Introduction

The amygdala, hippocampus, parahippocampal regions and their interconnected neural circuits have been implicated in the fear memory consolidation processes (Kim and Fanselow, 1992; Kjelstrup et al., 2002; Frankland et al., 2006; Botterill et al., 2014). Lesions to these brain regions have also been found in patients with temporal lobe epilepsy (TLE; Quesney, 1986; Hudson et al., 1993; Wolf et al., 1997; Bernasconi et al., 2003; Gyimesi et al., 2010; Meletti et al., 2014) and in animal models of TLE (Schwob et al., 1980; Ben-Ari, 1985; Pitkänen et al., 1998; Brandt et al., 2006; Toyoda et al., 2013). Moreover, patients with TLE and animal models of TLE with such lesions exhibit interictal emotional disturbances and impaired emotional learning that are known to be amygdala- and/or hippocampus-dependent (Hort et al., 1999; Vazquez and Devinsky, 2003; Kemppainen et al., 2006; Cardoso et al., 2009).

The pilocarpine-induced mouse model of TLE is commonly used for modeling TLE in humans. These epileptic mice share similar neuropathological mechanisms and seizure states with human TLE; animals experiencing status epilepticus (SE) for several hours showed histopathological alterations throughout the limbic system (Coulter, 1999; Curia et al., 2008; Tang and Loke, 2010; Reddy and Kuruba, 2013). The affected areas involve not only the hippocampus but also the amygdala, the entorhinal cortex (Du et al., 1995; Biagini et al., 2005; Wozny et al., 2005; Ma et al., 2008), the piriform cortex, the thalamus (Mathieson, 1975; Mello and Covolan, 1996), and specifically the midline thalamic nuclei (including the medial dorsal nucleus; Ben-Ari et al., 1980; Lothman and Collins, 1981; Cavalheiro et al., 1991). Although these affected areas are directly connected to the hippocampus and are similar to those found in human TLE, it is still unclear if there are any lesions in other regions not observed in human TLE. Therefore, it is possible that the extensive brain lesions in the pilocarpine-induced epilepsy mouse model are not necessarily related with the TLE condition *per se*. Nonetheless, the epilepsy mouse model induced by pilocarpine is still a valuable tool for understanding the pathological mechanisms of epilepsy in general as well as to determine the suitability of this mouse model as an experimental model of TLE (Curia et al., 2008). In addition to the standard epilepsy or TLE mouse model used here, there are also modified models considering the appropriate SE duration and the methods of SE induction in animals to reproduce the most extensive characteristics of human TLE (Depaulis and Hamelin, 2015).

Lesions in the basolateral amygdala (BLA) region abolished conditional freezing in response to contextual cues associated with foot shocks, suggesting that BLA is important in mediating conditioned fear responses (Maren et al., 1996; Fanselow and LeDoux, 1999; Vazdarjanova and McGaugh, 1999). There are also studies that have shown that damage to the BLA alone did not result in deficits in contextual or auditory fear memory (Goosens and Maren, 2001; Rabinak et al., 2009). Thus, the involvement of BLA in contextual fear memory is still unclear. We previously reported substantial neuronal damage in the CA1–3 fields of the hippocampus (Ma et al., 2006; Tang et al., 2006) in SE animals. There are also studies from other groups using the same standard or modified mouse epilepsy model that showed impaired extinction of fear and maintained amygdala-hippocampal theta synchrony (Lesting et al., 2011); c-Fos, JunD and HSP27 immunoreactivity in different parts of the brain following SE (Dubé et al., 1998); impairs spatial memory (Detour et al., 2005); and depressive impairments (Pineda et al., 2010). However, the organization of the neural circuitry of the BLA and subiculum in contextual fear memory in these SE animals is still unknown.

Herein, we used the SE mouse model to specifically examine the involvement of the subiculum and BLA in fear memory. The correlation between c-Fos protein expression in the hippocampus and the amygdala, and the behavioral changes, determines if these regions play a role in contextual fear learning and memory. We also examined the reorganization of the BLA and the subiculum pathway in epilepsy, by morphological analysis following the iontophoretical injection of an anterograde tracer, PHA-L or a retrograde tracer, cholera toxin B subunit (CTB) into the BLA.

## Materials and Methods

### Pilocarpine Treatment

Handling and care of all animals were in compliance with the NIH guidelines for animal research. All animal experiments were approved by the Institutional Animal Care and Use Committee at the Tan Tock Seng Hospital/National Neuroscience Institute. Male Swiss mice (25–30 g) used for all experiments in this study were treated as described previously (Ma et al., 2006, 2008; Tang et al., 2006). In brief, mice were given a single subcutaneous injection of methyl-scopolamine nitrate (1 mg/kg) 30 min before the injection of either saline or pilocarpine to limit the peripheral toxic effects. Mice in the pilocarpine—injection group received pilocarpine (300 mg/kg) through a single intraperitoneal injection. These mice exhibited behavioral changes such as tremor, mild facial clonus, head bobbing, hypoactivity and myoclonic movements of the limbs for about 4 h. These symptoms eventually progressed to recurrent myoclonic convulsions with falling, rearing and SE. The mean latent period from pilocarpine treatment to the first seizure observed is typically around 14 days. Racine stages (Racine, 1972) were used for the scoring of convulsive behavior, as follows: stage 0, no reaction; stage 1, mouth and facial movements such as eye blinking, stereotypic mouthing and/or mild facial clonus; stage 2, signs of severe facial clonus and/or head nodding; stage 3, involuntary myoclonic jerks in the forelimbs; stage 4, clonic convulsions in the forelimbs with rearing; and stage 5, generalized motor convulsions typically associated with the loss of balance. Mice at 2 months after pilocarpine-induced SE were designated as SE2m groups.

### Iontophoretical Injection of PHA-L or CTB

A total 50 mice were used (17 injected with saline and 33 injected with pilocarpine). Twenty-one of thirty-three pilocarpine-injected mice survived (mortality rate: 36.36%) at two months after injection (SE2m). These 17 saline-injected and 21 pilocarpine-injected mice were randomly allocated to four experimental groups: (1) control with PHA-L (*n* = 8); (2) SE2m with PHA-L (*n* = 11); (3) control with CTB (*n* = 9); and (4) SE2m with CTB (*n* = 10). Four control mice (2 injected with PHA-L and 2 injected with CTB) and 7 SE2m mice (four injected with PHA-L and three injected with CTB) were excluded from data analysis due to incorrect injection sites.

For injection of tracers, mice were first anesthetized with chloral hydrate at a dose of 400 mg/kg. With the heads of the mice immobilized using a Stoelting stereotaxic apparatus, small holes were drilled through the skulls to form specific injection sites positioned 1.6 mm posterior to bregma, 3.3 mm lateral to midline and 4.2 mm ventral to the dura for the control mice and 1.4 mm posterior to bregma, 3.1 mm lateral to midline and 4.2 mm ventral to the dura for the epileptic mice. 10% CTB (List Biological Laboratories, CA, USA) in distilled water or 2.5% PHA-L (Vector Laboratories, Burlingame, CA, USA) in 0.1 M phosphate buffered saline (pH 7.4) in a glass micropipette with a diameter of 20–30 μm was delivered iontophoretically into the BLA using a positive current of 5 μA (7 s on, 7 s off) over 10 min. Animals were sacrificed at 7 days after CTB or PHA-L injection and were perfused transcardially using 50 ml of saline, 100 ml of 4% paraformaldehyde and 0.15% picric acid in 0.1 M PB at pH 7.4 for 30 min. Coronal brain sections of 40 μm thickness were cut using a cryostat and the serial brain sections were collected in individual wells of 24-well plates.

### Immunocytochemical Studies

For immunohistochemistry, free-floating brain sections were first washed with TBST (0.1 M Tris buffered saline (TBS) containing 0.1% Triton-X 100). Brain sections were then incubated overnight at 4°C in mouse anti-NeuN antibody (1:1000; Chemicon International, Inc., CA, USA), goat anti-c-Fos antibody (1:1000; Santa Cruz Biotechnology, CA, USA), goat anti-PHA-L antibody (1:5000; Vector Laboratories, Burlingame, CA, USA), or goat anti-CTB antibody (1:2000; List Biological Laboratories, CA, USA), then washed three times with TBST before incubation in their respective secondary antibodies diluted 1:500 with TBST (biotinylated-horse anti-goat IgG for PHA-L or CTB; goat anti-mouse IgG for NeuN; or donkey anti-goat IgG for Fos for 2 h at room temperature). Brain sections were then incubated in avidin-biotin complex (ABC) reagent in TBST for 2 h, washed with TBS and allowed to react in a solution of 0.12% H_2_O_2_ and 0.05% DAB (Sigma-Aldrich, Missouri, USA) in TBS for 20 min, mounted on glass slides, covered with coverslips, and photographed using an image capture system. PHA-L stained sections were also counterstained with cresyl fast violet (CFV).

Double labeling of PHA-L with calbindin (CB), calretinin (CR) or parvalbumin (PV) was carried out by incubating brain sections in goat anti-PHA-L (1:1000), together with rabbit anti-CB (1:2000), anti-CR (1:1500) or anti-PV (1:1500) for 48 h at 4°C. The brain sections were washed and incubated in biotinylated horse anti-goat IgG (1:500) and swine anti-rabbit IgG (1:100) for 4 h at room temperature, then incubated in ABC solution for 2 h at room temperature before reaction with 3,3′-diaminobenzidine (DAB)-Nickel solution for 20 min. Subsequently, rabbit peroxidase anti-peroxidase (PAP; 1:100) solution was added to the brain sections and left overnight to incubate, and sections were then developed with DAB alone.

### Cell Counting and Data Analysis

Every third sections were stained with antibodies against NeuN or c-Fos for cell counting. BLA was determined anteroposterior (AP) to Bregma (AP: −1.40 mm; −1.90 mm; −2.30 mm). In the BLA, the total number of neurons per area in the specified regions of interest (ROI) in control mice and epileptic (SE2m) mice were quantified and the data were normalized with area shrinkage of SE animals in the specific brain regions. The c-Fos-expressing neurons in specific brain regions such as the hippocampus (AP: −1.70 mm; −1.94 mm; −2.18 mm), the dorsal (dSub) and ventral (vSub) subiculum (AP: −3.28 mm; −3.40 mm; −3.52 mm), were also counted and presented as density (number of immunopositive neuron per square millimeter; No./mm^2^) normalized with cell loss/area of SE animals as mean value ± standard deviation (SD).

PHA-L-immunopositive terminal-like structures in the vSub were visualized at high (400×) magnification (10× eyepiece magnification plus 40× objective lens) using a Zeiss wide field light microscope. These terminal-like structures were identified based on their visibly enlarged structures seen on PHA-L-immunopositive fibers. The numbers of these enlarged structures on fibers per mm^2^ (No./mm^2^) at specific ROI were counted and the data were normalized with area shrinkage of SE animals. ROI is defined as the specific brain region of interest (e.g., v-Sub) based on their anatomical locations in the brain. Images of the ROI were taken at the focal planes of the ROI that showed clearest and in-focused structures. The numbers of CB, CR or PV-immunopositive neurons showing physical contacts with the PHA-L-labeled terminal-like structures were also counted as described above. In the vSub, CTB labeled neurons were counted and presented as density (No./mm^2^) normalized with the area in SE animals relative to the control animals. Imaging-Pro Plus (Mediacybernetics, MD, USA) was used for all quantitative analysis in this study. The neuronal profile counts were blinded. Statistical analysis were done between the control and the SE2m mice at different time points using one-way ANOVA followed by Student-Newman-Keuls *post hoc* tests (for experiments with more than two groups of data). For comparing experiments with two groups of data, an independent samples test (*t-test*) was used instead. A *p*-value of less than 0.05 is considered statistically significant.

### Contextual Fear Memory Test

Fear conditioning and retention memory tests were conducted using a Y-maze with stainless-steel grid floor (30 × 10.5 × 14.5 cm) as described previously (Vazdarjanova and McGaugh, 1999). The walls of the shock arm were decorated with red wallpaper and two small red neon lights were installed on the floor. The walls of the other two non-shock arms were decorated with gold and silver wallpaper with two small green neon lights installed on the floor. The floor of the shock arm was connected to an AC shock generator controlled by a timer. A video camera mounted 1.2 m above the maze was used to monitor the behavioral changes of each mouse.

For behavioral experiments, a total of 42 mice were used (12 for saline-injection and 30 for pilocarpine-injection). Twenty out of the thirty pilocarpine-injected mice survived (mortality rate: 33.33%) at 2 months after injection (SE2m). Four experiment groups were designed for behavioral test that included control/context group (N/C, *n* = 6), status epilepticus/context (SE/C, *n* = 9) group, control/context with foot shock group (N/CS, *n* = 6) and status epilepticus/context with foot shock group (SE/CS, *n* = 11). Each mouse was handled for 1 min per day for 3 days before the start of training to reduce stress in the later training and test. The arms were thoroughly cleaned with 10% ethanol solution directly after each training and testing session. On Day 1 (habituation day), mice were allowed to explore Y-maze freely for 8 min. The total numbers of entrance to each arm were recorded. On Day 2 (training day), mice in N/CS and SE/CS groups were placed in the shock arm that was blocked off from the rest of the maze. After 120 s, mice in the above two groups received four foot shocks each (0.3 mA AC, 1 s through stainless-steel grid delivered at 60 s intervals). Mice were returned to the home cages 1 min after the last foot shock. The total freezing times of each animal after each foot shock were recorded. The freezing time for each session was presented as a percentage of total observation. Mice in N/C and SE/C groups were placed in the shock arm for the same period of time, without receiving foot shocks. On Day 3 (test day), mice were placed in a non-shock arm and allowed to access all arms of the maze for 4 min. The total freezing time and latency to enter shock or another non-shock arm were recorded. The percentage of the number of the shock arm entries to all arm entries was calculated. No shock was delivered on Day 3.

For statistical analysis of behavioral data, we used repeated-measures-ANOVA to assess the effects of the lesion and foot shock treatments on the freezing during training. The number of entries, freezing time, first entry into the shock arm, foot shock response and the time spent per arm of different experimental groups were also compared using ANOVA. All *post hoc* comparisons were done using Fisher’s tests. All probabilities less than 0.05 are considered significant.

For immunohistochemistry studies, these animals were sacrificed 1 h after the behavioral test.

## Results

### Neuronal Loss in the BLA and vSub of Epileptic Mice

**Figure 1:**
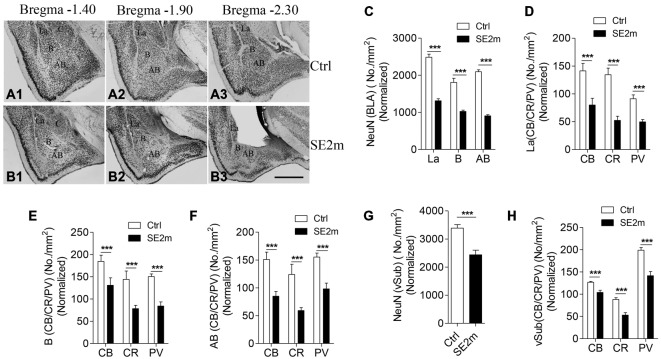
**Neuronal loss in the BLA and vSub of epileptic mice induced by pilocarpine treatment.** Immunocytochemistry for NeuN shows the lateral (La), basal (B) and accessory basal (AB) nuclei in BLA of control **(A1–3)** and epileptic mice **(B1–3)**. Bar graph **(C)** shows the number of neurons (NeuN positive) per area (mm^2^) in the different regions (La, B and AB) of the amygdala in epileptic mice (SE2m) normalized to the corresponding areas in control mice (Ctrl; Student’s *t*-test, ***p < 0.001). Bar graphs **(D–F)** show the number of CB-, CR- or PV-immunopositive neurons (Student’s *t-test*, ***p < 0.001) per area (mm^2^) at the La, B and AB nuclei of amygdala in epileptic and control mice. Bar graph **(G)** shows the number of NeuN positive neurons per area (mm^2^) in the vSub of epileptic and control mice (Student’s *t-test*, ***p < 0.001). Bar graph **(H)** shows the number of CB-, CR- or PV-immunopositive neurons per area (mm^2^; Student’s *t-test*, ***p < 0.001) at the vSub in epileptic and control mice. Scale bar = 200 μm in **(B3)** applies to **(A1–3)** and **(B1–2)**. The numbers of neurons per area in epileptic mice on all graphs were normalized to the corresponding areas in control mice.

The specific regions in BLA were determined and labeled according to previous studies on the amygdaloidal complex (Pitkänen et al., 1998; Sah et al., 2003). BLA comprises the lateral (La), basal (B), and accessory basal (AB) nuclei. The lateral nucleus is adjacent to the basal nucleus ventrally and is located dorsally. This region is bordered medially by the central nucleus (Ce) and laterally by the external capsule. The basal nucleus is located ventral to the lateral nucleus and is subdivided into the more caudal intermediate and parvocellular subdivisions, and the rostral magnocellular subdivision. The AB nucleus lies adjacent to the amygdalohippocampal area and is ventral to the basal nucleus (Figures 1A,B).

Quantitative analysis showed that the areas of the La, B and AB nuclei of the BLA are 0.1178 ± 0.0158, 0.2069 ± 0.0151 and 0.1420 ± 0.0115 mm^2^ in the epileptic mice (SE2m) and 0.1514 ± 0.0198, 0.2396 ± 0.032 and 0.1894 ± 0.0153 mm^2^ in the control mice respectively. There were no significant differences in the La and B nuclei areas (Student’s *t*-test, n.s. *p* > 0.05) between the epileptic and control mice. However, a significant decreased in the AB nucleus area (Student’s *t-test*, *p < 0.05) was observed in the epileptic mice as compared to the control mice. Therefore, to account for this atrophy in the epileptic mice, total neurons per area in the epileptic mice were normalized to the same area of the control mice. NeuN immunocytochemistry showed 47.15, 43.26 and 56.80% reduction in total number of neurons at the La, B and AB nuclei of the amygdala, respectively, in the epileptic mice (SE2m) as compared to the control mice (Figures 1A–C). The populations of CB-, CR- and PV-immunopositive neurons in the epileptic mice were significantly decreased (Student’s *t-test*, ***p < 0.001) at the La (48.24, 61.05, and 36.66% reduction), B (26.56, 45.59, and 44.04% reduction) and AB (43.30, 52.27, and 36.79% reduction) nuclei of the amygdala (Figures 1D–F).

There was no significant area change (Student’s *t*-test, *p* > 0.05) in the vSub of epileptic mice (0.7233 ± 0.0617 mm^2^) as compared to control mice (0.6200 ± 0.0584 mm^2^). NeuN immunocytochemistry showed a significant 28.06% reduction in total number of neurons at the vSub in the epileptic mice as compared to the control mice (Figure 1G). The numbers of CB-, CR- and PV-immunopositive neurons were significant decreased by 17.80, 39.98 and 28.80% respectively (Student’s *t-test*, ***p < 0.001) at the vSub in the epileptic mice (Figure 1H).

### Pathological Changes of Afferent and Efferent Pathways from the BLA in Epileptic Mice

**Figure 2:**
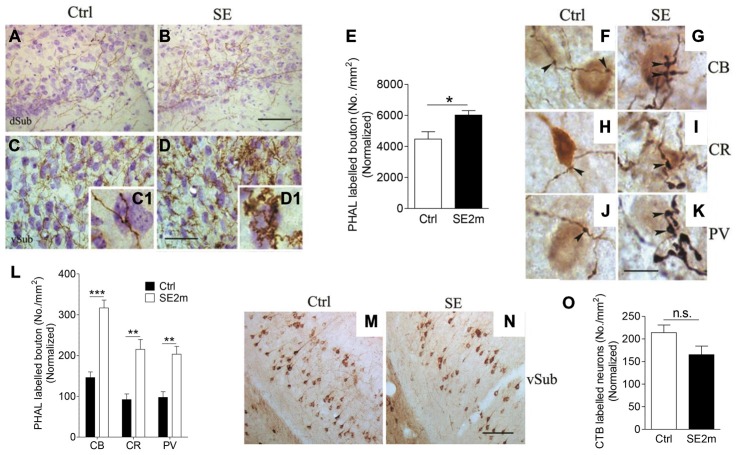
**Increased terminal-like structures in the vSub of epileptic mice.** Representative images showing PHA-L-immunopositive fibers in the vSub region of control **(A,C)** or epileptic **(B,D)** mice. Graph shows density of PHA-L-labeled terminal-like structures in both groups of mice normalized with area shrinkage in the SE2m group **(E)** (Student’s *t*-test, *p < 0.05). Representative images showing double immunostaining of CB, CR or PV-positive cells with PHA-L-immunopositive fibers in the control (**F**,**H** and **J** respectively) and epileptic (**G,I** and **K** respectively) mice. Graph shows quantification of the number of CB, CR or PV-immunopositive neurons contacted by PHA-L labeled en passant and terminal-like structures in vSub normalized with CB, CR or PV-immunopositive neuronal loss in the SE2m group **(L)** (Student’s *t-test*, ***p < 0.001, **p < 0.01). Representative images showing CTB-immunopositive neurons at the ipsilateral vSub in control **(M)** and epileptic **(N)** mice. Graph **(O)** showing the number of retrograde-labeled neurons per area in the ipsilateral vSub of control and epileptic mice normalized with neuronal loss in the SE2m group (Student’s *t-test*, n.s. *p* > 0.05). Scale bar = 100 μm in **(B)** and **(N)** applies to **(A,B)** and **(M,N)**. Scale bar = 50 μm in **(D)** applies to **(C,D)**. Scale bar = 10 μm in **(K)** applies to **(F–J)**.

PHA-L was iontophoretically injected into the BLA in control or epileptic mice and these mice were observed for PHA-L labeling in the vSub (Figures 2A,B). Images taken at higher magnification showed PHA-L-immunopositive fibers and terminal-like structures (visible under high magnification (Figures 2C1,D1) in the vSub of control and epileptic mice (Figures 2C,D) indicating possible contact of neurons at BLA with neurons at vSub. The density of terminal-like structures on PHA-L-immunopositive fibers was also significantly higher (25.63% increase) in the vSub of epileptic mice (average of 6019 terminal-like structures/mm^2^) compared to that of control mice (average of 4476 terminal-like structures/mm^2^; Figure 2E; Student’s *t*-test, *p < 0.05).

To determine the contact points of PHA-L-immunopositive en passant and terminal-like structures in vSub, we carried out double immunostaining of PHA-L with CB, CR or PV. We found visible contacts between PHA-L immunopositive en passant and terminal-like structures and CB, CR or PV-immunopositive neurons in vSub of mice in both the control (Figures 2F,H,J) and epilepsy group (Figures 2G,I,K). Quantitative assessment showed an increased number of CB, CR or PV immunopositive neurons in the vSub contacted by PHA-L-labeled en passant and terminal-like structures (Figure 2L) in the epileptic mice as compared to the control mice (Student’s *t*-test, ***p < 0.001, **p < 0.01).

Next, we iontophoretically injected CTB into the BLA of control and epileptic mice to determine the retrograde connections from BLA. CTB-immunopositive neurons were found mainly in the ipsilateral vSub in both the control (Figure 2M) and the epileptic mice (Figure 2N). However, quantitative assessment shows that the number of retrograde labeled neurons in the ipsilateral vSub was not significantly different in the epileptic mice as compared to the control mice (Figure 2O; Student’s *t*-test, *p* > 0.05).

### Impaired Learning and Memory Function in Epileptic Mice

**Figure 3:**
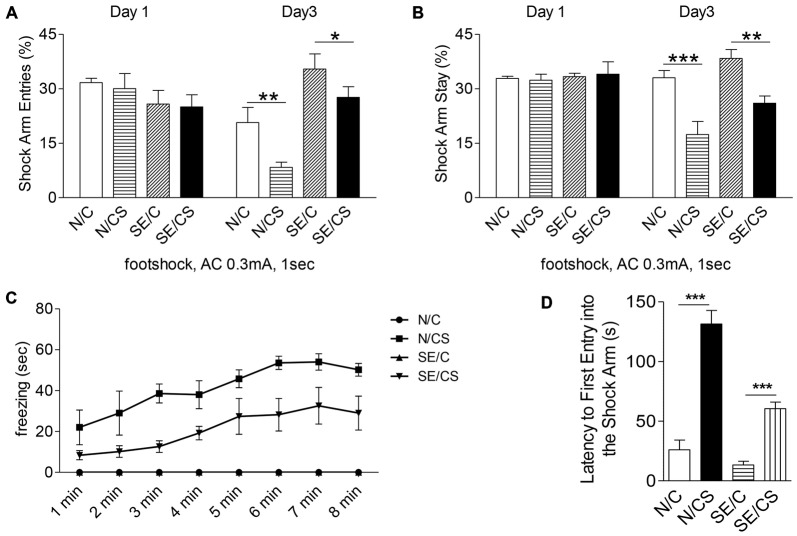
**Pilocarpine-induced epilepsy in mice impairs fear memory.** Graphs showing the percentage of shock arm entries **(A)** and shock arm stay **(B)** of the four groups of mice (N/C—control mice, no foot shocks; N/CS—control mice, with foot shocks; SE/C—SE mice, no foot shocks; SE/CS—SE mice, with foot shocks) on habituation day (day 1) and on test day (day 3). Graph showing the freezing time (sec) in control or SE mice with (N/CS and SE/CS groups) or without (N/C and SE/C groups) foot shocks on day 2, when a series of four foot shocks was given **(C)**. Graph shows the latency of first entry into the shock arm of the four groups of mice as indicated **(D)** (ANOVA *post hoc* test, ***p < 0.001, **p > 0.01, *p < 0.05).

We next investigated if the structural changes in epileptic mice were associated with functional consequences. Since epilepsy has been shown to affect hippocampal functions, we selected Y-maze contextual fear memory test to assess learning and memory function in the control and epileptic mice. During habituation (Day 1), mice in all groups explored all three arms of the maze evenly and displayed no freezing. There were no differences in the percentage of shock arm entries (Figure 3A, Day 1) or shock arm stay (Figure 3B, Day 1) among the groups of mice. On Day 2, when a series of four foot shocks were given, the freezing time increased in both control and epileptic mice (N/CS and SE/CS groups; Figure 3C). N/C and SE/C group not subjected to foot shocks did not exhibit any freezing response (Figure 3C). Further analysis with one-way ANOVA followed by Newman-Keuls *post hoc* multiple comparisons and a paired *t*-test indicated that the N/CS group exhibited significantly longer freezing times after each of the four foot shocks as compared to the SE/CS, SE/C and N/C groups (ANOVA *post hoc* test, ***p < 0.001; Figure 3C). There was no significant difference in the percentage of freezing time between SE/C and N/C groups (*p > 0.05), but SE/CS group showed significantly longer freezing times than N/C and SE/C groups after each of the four shocks (ANOVA *post hoc* test, ***p < 0.001).

On Day 3, all mice explored the shock arm, but the N/CS group showed longer latency to enter the shock arm than the SE/CS, N/C and SE/C groups (ANOVA *post hoc* test, ***p < 0.001, Figure 3D). SE/CS group showed longer latency to enter the shock arm compared to the N/C and SE/C groups (ANOVA *post hoc* test, ***p < 0.001, Figure 3D). The percentage of shock arm entries to that of all arm entries was significantly lower in the N/CS group than the N/C group (ANOVA *post hoc* test, **p < 0.01) and the SE/CS group compared to the SE/C group (ANOVA *post hoc* test, *p < 0.05; Figure 3A, Day 3). The percentage of the shock arm stay to that of all arm stay was significantly lower in the N/CS group compared to N/C group (ANOVA *post hoc* test, ***p < 0.001) and SE/CS group than SE/C (ANOVA *post hoc* test, **p < 0.01; Figure 3B, Day 3).

### Memory Test Enhances c-Fox Expression in Epileptic Mice

**Figure 4:**
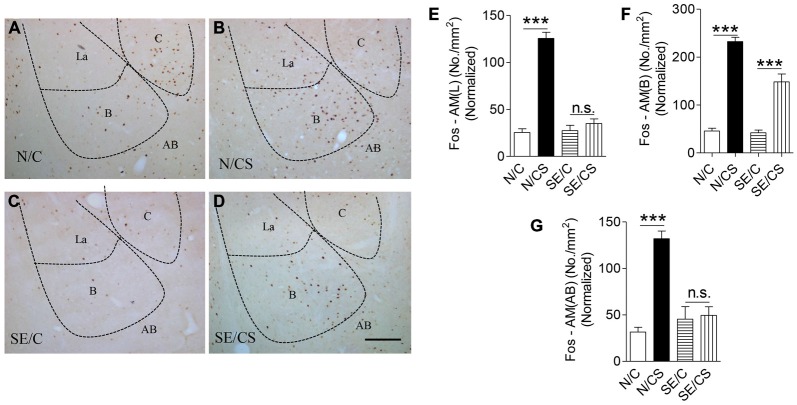
**Increased expression of c-Fos at the BLA upon contextual learning and memory test.** Representative images show c-Fos immunopositive neurons in the amygdala of the four groups of mice after fear memory test at Day 3 **(A–D)**. Graphs **(E–G)** showing the number of c-Fos-immunopositive cells per area (mm^2^) in the lateral **(E)** basal **(F)** and accessory basal **(G)** nuclei of the amygdala after fear memory test at Day 3 in the four groups of mice normalized with neuronal loss in the SE2m group (ANOVA *post hoc* test, ***p < 0.001), Scale bar = 100 μm in **(D)** applies to **(A–C)**. La, Lateral; B, Basal; C, Central; AB, Accessory Basal.

c-Fos is required for hippocampal functions (Guzowski et al., 2001). We found a significantly higher density of c-Fos-immunopositive neurons in the N/CS group (Figures 4A–D), specifically in the lateral (Figure 4E), basal (Figure 4F) and AB (Figure 4G) nuclei of the amygdala after the fear memory test at Day 3 (ANOVA *post hoc* test, ***p < 0.001) as compared to the N/C, SE/C and SE/CS groups (Figures 4A–D). More c-Fos-immunopositive neurons were observed in the basal nucleus of SE/CS group than those in SE/C group (ANOVA *post hoc* test, ***p < 0.001; Figures 4A–D,F) indicating higher neuronal activity in these neurons upon fear conditioning. However, there are no significant changes in expression of c-Fos in the lateral (Figure 4E) and AB (Figure 4G) nuclei of the amygdala in SE/C and SE/CS groups (ANOVA *post hoc* test, *p* > 0.05).

**Figure 5:**
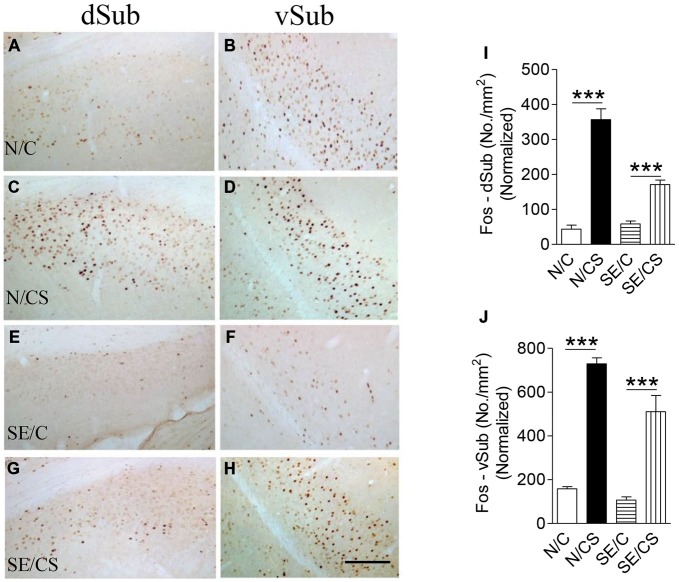
**Increased expression of c-Fos at the vSub upon contextual learning and memory test.** Representative images show c-Fos immunopositive neurons in the dorsal (dSub) **(A,C,E,G)** and ventral (vSub) **(B,D,F,H)** subiculum in the different groups as indicated. Graphs **(I,J)** show quantification of c-Fos-immunopositive neurons per area (mm^2^) in dSub **(I)** or vSub **(J)** normalized with neuronal loss in the SE2m group (ANOVA *post hoc* test, ***p < 0.001). Scale bar = 100 μm in **(H)** applies to **(A–G)**.

The density of c-Fos-immunopositive neurons in N/CS group was increased significantly in the dorsal (dSub; Figures 5C,I) and ventral (vSub; Figures 5D,J) subiculum (ANOVA *post hoc* test, ***p < 0.001) after the fear memory test at Day 3 (ANOVA *post hoc* test, ***p < 0.001) as compared to those in N/C (Figures 5A,B,I,J), SE/C (Figures 5E,F,I,J) and SE/CS groups (Figures 5G–J). However, more Fos-immunopositive neurons in both areas (Figures 5E–J) were observed in SE/CS group as compared to those in SE/C group (ANOVA *post hoc* test, ***p < 0.001).

## Discussion

### Pilocarpine-Induced SE Mouse to Study Anatomical Changes in Different Brain Regions in Epilepsy

Pilocarpine-induced SE, when not interrupted by drug administration, is able to induce reorganization and anatomical damage in brain regions such as the olfactory cortex, amygdala, thalamus, hippocampus and neocortex (Curia et al., 2008; Reddy and Kuruba, 2013). Pharmacological intervention, such as diazepam, has been used to limit the SE duration to reduce mortality as well as to confine neuronal damage to the hippocampal formation and related limbic regions. One study showed Fluorojade-positive neurodegeneration in near 30 brain regions with several of these regions well beyond the temporal lobe in animals with 90 min of SE induced by either systemic or intra-hippocampal pilocarpine aborted by diazepam (Castro et al., 2011), However, the pilocarpine-induced SE model used here without pharmacological intervention is still widely used for studying pathological mechanisms underlying epilepsy in general because animals with shorter SE duration limited by drug treatment showed a higher possibility of not developing spontaneous recurrent seizures (SRSs).

### Cytoarchitectonic Changes in the BLA of Epileptic Mice

Neuronal loss and gliosis in the amygdala have been reported in a large number of neuropathological studies using animal TLE models and in patients with TLE (Turski et al., 1983; Callahan et al., 1991; Wolf et al., 1997; Pitkänen et al., 1998; Klitgaard et al., 2002; Bernasconi et al., 2003; Brandt et al., 2006; Toyoda et al., 2013; Meletti et al., 2014). However, there is still no detailed and thorough study on the neuropathological changes in the amygdala of epileptic mice. Herein, we observed substantial neuronal loss in the BLA in these epileptic mice that exhibit deficits in contextual fear memory. These data suggested that epilepsy affected the structure of amygdala and the amygdala-dependent fear memory. However, the BLA remained relatively undamaged (~66–76% of neurons), possibly providing residual connections for fear-related behaviors.

### Behavior Changes and c-Fos Activation in Epilepsy

c-Fos is an immediate early gene. It is part of the activator protein-1 transcription factor and has been postulated to participate in the molecular mechanisms of learning and memory. c-Fos was selected as a marker of neuronal activity because it has been used to assess region-specific activity during fear conditioning in previous studies (Campeau et al., 1991; Beck and Fibiger, 1995; Rosen et al., 1998; Majak and Pitkänen, 2003; Botterill et al., 2014). Our current observations and previous studies showed a time-dependent increase in c-Fos protein in the hippocampus, amygdala and other related regions. This increased in expression occurred 30–90 min post training in memory test, with expression peaking at 60 min, and returning to basal levels around 4 h (Dragunow and Faull, 1989; Tischmeyer and Grimm, 1999). Therefore, immunohistochemical assays for c-Fos expression were done 1 h after the behavioral test.

The shock-trained epileptic mice in our study entered the shock arm with shorter latencies and at higher frequencies as compared to the shock-trained control. This indicates that fear memory of epileptic mice was significantly impaired. However, we still observed increased c-Fos expression in the dentate gyrus of epileptic mice. This increase in amygdala c-Fos expression and that epileptic mice with significant neuronal loss in the hippocampus could still demonstrate freezing responses upon entering the shock arm suggest that another neural pathway is involved in contextual fear memory. The relatively undamaged BLA in the epileptic mice could potentially still play a role in fear memory even when the hippocampus is substantially damaged. Moreover, increased c-Fos expression, particularly in the BLA of control mice upon contextual fear memory test provided further evidence on the involvement of the BLA in foot shock dependent-fear memory. These results are in agreement with previous reports showing that cues-associated foot shock increases c-Fos mRNA and protein expression in the amygdala (Campeau et al., 1991; Beck and Fibiger, 1995; Rosen et al., 1998; Majak and Pitkänen, 2003; Botterill et al., 2014).

### Alteration of Neural Connections and Learning and Memory in Epilepsy

Here, we report that epileptic mice have significantly higher numbers of CR, CB or PV-immunopositive cells contacted by PHA-L labeled en passant and terminal-like structures in the v-Sub as compared to control mice. These connections are important for synaptic excitability and plasticity in the efferent and afferent pathways of BLA. The presence of ~50% more terminal-like structures in the v-Sub of epileptic mice that were labeled with PHA-L injected at BLA may provide the neuroanatomical basis to support the enhancement of interaction between BLA and v-Sub in epilepsy. These findings suggest an enhanced or compensatory higher excitability of neurons in the amygdala of epileptic mice.

Our data suggest that an increase in aberrant network connections and compensatory mechanisms in the parahippocampal formation may contribute to neurological deficits in the epileptic mice and possibly, also in humans with TLE. Therefore, a panoramic analysis beyond the temporal lobe of these epileptic mice is essential for better understanding the lesions and compensatory reorganization of circuitry in epilepsy. Currently, reorganizations of the neural circuits in several brain regions of this mouse epilepsy model including the hippocampus (Ma et al., 2006; Zhang et al., 2009), the subiculum (Tang et al., 2006; He et al., 2010) and the lateral entorhinal cortex (Ma et al., 2008) have been reported. Following these studies and our observations here on the amygdala, it will be of interest to analyze the reorganizations of other neural circuits directly connected with the hippocampus in epileptic mice, such as the v-Sub-retrosplenial cortex (RSC) circuits, perirhinal cortex (PRh)-hippocampus and PRh-entorhinal cortex (EC)-hippocampus circuits as well as prefrontal cortex-reuniens thalamic nucleus-hippocampus circuits.

## Conflict of Interest Statement

The authors declare that the research was conducted in the absence of any commercial or financial relationships that could be construed as a potential conflict of interest.
